# Mr. Purton, on the Operation for the Hare-Lip

**Published:** 1800-10

**Authors:** T. Purton

**Affiliations:** Alcester


To the EDITORS of the MEDICAL and PHYSICAL JOURNAL.

Gentlemen,

I Am induced, from the attention which you have already paid to my Communications, to offer a few Remarks on the Operation for the Hare-lip. I have often thought that this operation has been deferred too long after birth; and that if it was performed formed earlier, the child would not only ſuffer leſs, but there would be leſs difficulty in the operation. The reſult has fully anſwered my expectations: I find many advantages in the early performance of the operation; the principal are, the paſſive ſtate of the little patient, and the thinneſs of the lips. The common ſuture I think preferable to the pins, which, with a proper bandage to keep up the cheeks, is all that is neceſſary. I operate as early as the third week; and on the fourth day from the operation, the ligatures may generally be removed: but reſpecting this, there muſt neceſſarily be variety in different caſes.

Theſe remarks may be conſidered by ſome as trifling; but if they tend in the leaſt to diminiſh the ſufferings of the child, and at the ſame time give leſs impediment to the operator, I think you will deem them worthy of notice.

I am, Gentlemen,

Your very humble ſervant,

*Alcester, Sept.* 12, 1800. T. PURTON.

